# Utilising a Novel Virtual Reality System for Orthopaedic Pre-operative Trauma Planning

**DOI:** 10.7759/cureus.91198

**Published:** 2025-08-28

**Authors:** Dominic Waugh, Rahul Bhattacharyya, Oliver Bailey, David Howie

**Affiliations:** 1 Trauma and Orthopaedics, NHS Greater Glasgow and Clyde, Glasgow, GBR; 2 Trauma and Orthopaedics, NHS Lanarkshire, Glasgow, GBR

**Keywords:** health tech innovations, orthopaedic, plan evaluation, trauma imaging, virtual reality (vr)

## Abstract

With increasing trauma burden, there is a need to improve the efficiency of care while improving surgical accuracy to minimise complications. Common fracture operations have been shown to have high failure rates, often related to implant-related technical errors. Pre-operative surgical planning has been shown to enhance orthopaedic surgical accuracy, particularly with robotic systems in elective arthroplasty. Historically, orthopaedic surgical planning was performed using plain radiographs, although modern three-dimensional (3D) techniques have been shown to be superior for assessing complex fracture patterns and are now in more routine use. While virtual reality (VR) environments have demonstrated benefits in delivering surgical training, there is little in the literature to describe surgeons' use of VR to visualise both patient-specific computed tomographic (CT) anatomy and surgical implants in the same virtual space. It was the author's aim to source and build an affordable system that would allow visualisation of 3D patient-specific CT data with the additional ability to augment this model with surgical implants, facilitating a full 3D trauma visualisation and planning system for complex trauma for surgical planning. Patient CT images were obtained as part of routine clinical care and viewed on local hospital patient imaging systems. An existing computer was upgraded with a graphics card to run additional software from Medicalholodeck™ (MedicalImaging XR™, Zurich, Switzerland). A VR gaming headset was purchased to utilise the system. A non-disclosure agreement was signed with our trauma implant supplier to allow access to virtual trauma implant object files. Patient-specific images can be opened on local computer software and imported to the Medicalholodeck™ MedicalImaging XR™ VR environment. Patient-specific images can be manipulated in 3D to review fracture patterns. Implant-specific files can be opened and placed within the same VR environment to plan optimal fixation. Local information technology (IT) governance procedures were followed during procurement, and patients agreed to the use of images in publication and research. To our knowledge, we are the first to describe this relatively low-cost, high-fidelity VR system using Medicalholodeck™ software, allowing visualisation of stereoscopic 3D patient images with specific orthopaedic surgical implants loaded into the same virtual space for orthopaedic trauma. We have been able to demonstrate proof of functionality in what we believe is a very useful tool to aid trauma and orthopaedic pre-operative surgical planning and execution going forward. In the future, we will look to demonstrate perceived usefulness by the orthopaedic team for fracture understanding and trauma planning. It is plausible that a VR system allowing accurate templating for orthopaedic trauma surgery could be of great benefit to both surgeon and patient and may have an influence over such factors as operative time, failure of fixation, surgeon satisfaction, and patient outcomes.

## Introduction

Bone fractures pose a significant problem for global health systems. Recent data provided by the ORthopaedic Trauma Hospital Outcomes - Patient Operative Delays (ORTHOPOD) study group highlighted that over 70% of trauma cases operated on represented fractures, with 12% of operations cancelled at least once [1]. With increasing trauma burden, there is a need to develop novel ways to improve efficiency and accuracy to reduce the added burden of return to theatre due to inadequate pre-operative planning and subsequent failed fixation. Common operations such as fixation of intertrochanteric hip fractures have seen reported failure rates of between 3% and 12% [2], though rates can be higher in more complex trauma patients. Managing complex fracture patterns inevitably leads to increased risk for the patient and intensive use of health service resources. This group in particular should have appropriate preoperative planning to ensure the surgery is done by the right person, at the right time, and in the right way. Historically, fracture surgery assessment and planning were based on a two-dimensional (2D) radiograph, though the use of computed tomography (CT) three-dimensional (3D) imaging techniques has been demonstrated to have superior rates of fracture detection in areas such as ankle [3] and knee trauma [4]. CT scans are now routine for more complex intra-articular fractures. Even with the improvement in imaging techniques, technical errors in fracture surgery still occur. An analysis of 1,093 trauma patients from the Netherlands who underwent fracture fixation demonstrated an error rate of 8.7%, with up to 30% of these cases having potentially significant consequences [5]. Lower error rates do not indicate lesser consequences; an analysis of 3,758 Dutch patients reported an error rate of 1.8%; however, 85% of these required reoperation [6]. Of these patients, around two-thirds of the errors were due to incorrect implant positioning, use of the wrong implant, incorrect implant length, and incorrect use of the implant [6]. Pre-operative planning has been demonstrated to enhance orthopaedic surgical accuracy, with robotic systems demonstrating improvements in component implantation (in non-trauma arthroplasty) compared to other orthopaedic techniques [7]. In trauma surgery, there is further potential to reduce complications and improve surgical outcomes, such as with 3D-assisted surgery of tibial plateau fractures, which has been shown to reduce both operative time and blood loss [8]. While the risk of error could be improved with appropriate preoperative planning, traditional systems have inherent limitations. Viewing patient CT imaging is typically limited by the use of a 2D monitor to visualise the 3D construct. There is evidence that the use of a 3D monitor with polarised glasses has significant advantages when compared to those in 2D, with enhancements of depth perception and spatial awareness, allowing more accurate representation of three-dimensional anatomical structures and improving performance in laparoscopic surgery [9]. While the use of 3D virtual reality (VR) environments has been demonstrated to improve preoperative surgical planning in multiple specialities, such as for robotic partial nephrectomy [10], the inability to concurrently view CT images with trauma implants further inhibits surgeons' ability to accurately template preoperatively in trauma surgery using systems we have access to currently within the United Kingdom National Health Service (UK NHS).

VR describes an exclusively digitised environment in which the wearer’s view of the real world is obstructed. These systems provide the ability for users to interact with an artificial, computer-generated environment with hand-operated controllers to augment the interactive experience [11]. Studies have demonstrated that VR-based training can lead to accelerated technical skill acquisition, such as in arthroscopy with relation to camera movement, anatomy recognition and instrument triangulation [12]. Benefits have also been seen in practising open surgical techniques; when undertaking intramedullary tibial nail insertion on sawbones, VR has been shown to increase procedural completion rates and accuracy [13]. Wider literature attention has demonstrated the potential for VR-assisted pre-operative planning to facilitate reductions in operating time, rates of blood loss, re-operations, re-admissions and overall morbidity while improving fracture reduction quality [14]. Other sectors have shown VR rehearsal as a useful training adjunct; VR has been suggested to enhance situational awareness in military decision-making [15], improve human-robot interactions within the construction sector [16] and reduce technical error rates within complex industrial maintenance tasks [17]. The use of CT data to generate 3D stereoscopic images is a relatively new technique, with technological advances allowing manipulation of volumetric medical images in VR. Multiple benefits of this technique have been reported when compared to conventional 2D images, including an increased perception of distance and depth by radiologists [18]. There is relatively little data indicating surgeon preference between the use of standard 2D techniques for fracture pattern understanding versus the use of VR to review 3D images. However, a usability study has demonstrated surgeon preference to use both a desktop viewer with 3D capabilities, along with a VR solution when visualising virtual models [19]. While using patient-specific geometrical models has been described in the literature, there is little published on VR as a pre-operative tool in trauma and orthopaedic surgery, where surgical implants can be manipulated within the same virtual environment as the CT data. It was the lead author's aim to find an affordable, high-fidelity VR system that would allow visualisation of stereoscopic 3D patient images with the additional ability to augment this model with surgical implants, facilitating a full 3D trauma visualisation and planning system for complex trauma.

## Technical report

**Figure 1:**
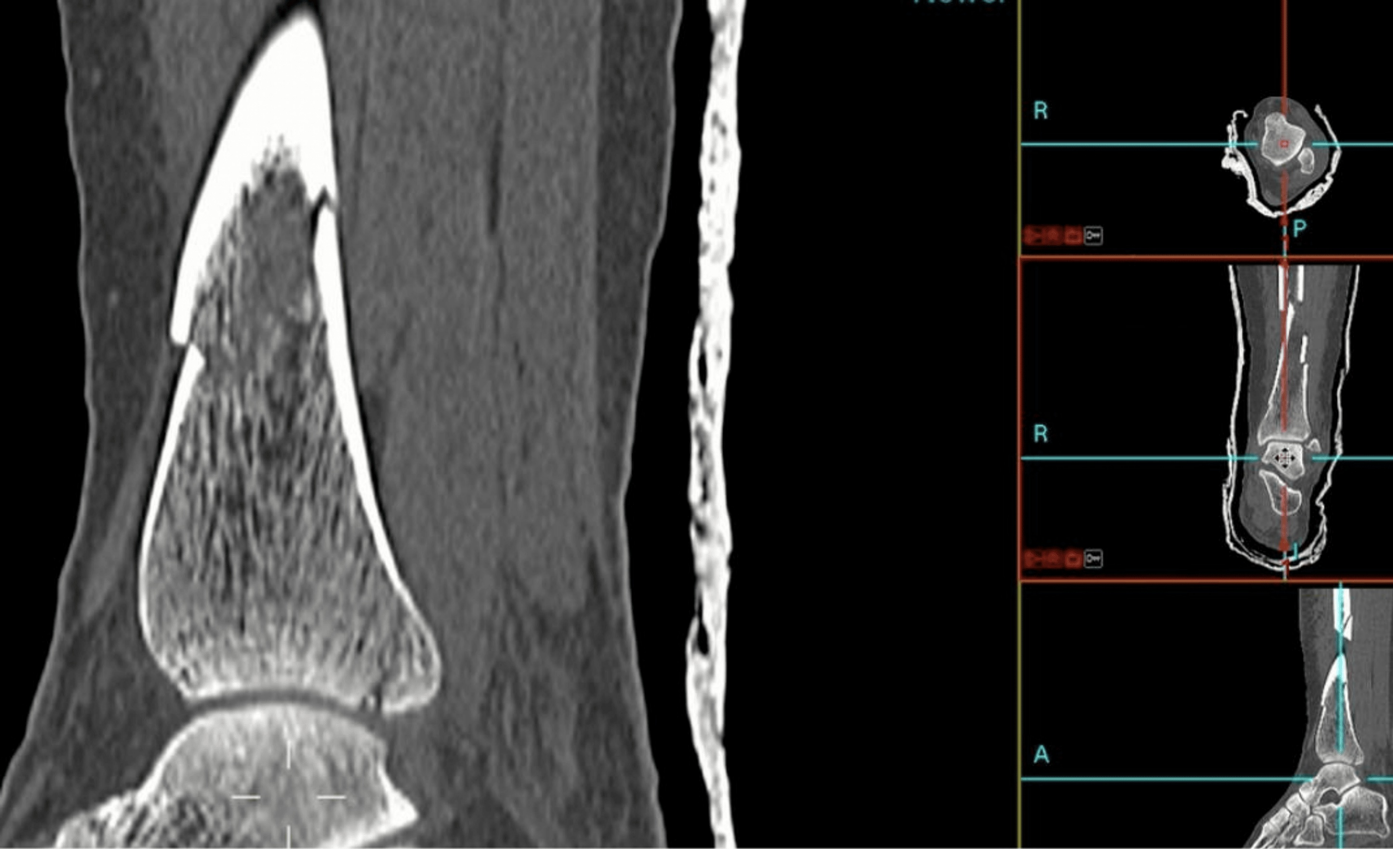
MPR DICOM images of a tibial shaft fracture with a posterior malleolus fragment MPR: multi-planar reconstructions; DICOM: Digital Imaging and Communications in Medicine This figure has been sourced from the "LOVE" (Lanarkshire Orthopaedic Virtual Environment) system, the in-house system at NHS Lanarkshire. Reproduced with permission from Medicalholodeck and Stryker.

Digital Imaging and Communications in Medicine (DICOM) file format is an established digital protocol used in the storage and sending of medical images, facilitating communication between hardware and software programs involved in hospital patient imaging systems. DICOM file format CT images obtained as a part of routine clinical care were viewed locally using Picture Archiving and Communication Systems (PACS) software (Kodak, Carestream Health, ONEX Corp., Rochester, NY, USA). Images were viewed and stored on local systems to ensure General Data Protection Regulation compliance. Patient DICOM images can also be visualised in PACS as 3D multi-planar reconstructions (MPR) to facilitate review [20], as demonstrated in Figure 1.

VR software platforms commercially available were explored and assessed for their ability to load DICOM data and STL files, and for cost. Only one was found to meet all our use case criteria: Medical Imaging XR™; Medicalholodeck® Inc. (Zurich, Switzerland). Our current trauma supplier was able to provide us with access to their implant library in .STL file format (Stryker, Kalamazoo, MI, USA) after signing a non-disclosure agreement. STL files comprise a series of linked triangles that describe the surface geometry of a 3D object and are commonly used in 3D printing and computer-aided design. Several uses of the STL file have been described with relation to 3D printing in trauma and orthopaedics, including tissue and organ fabrication, creation of customised prostheses, and pharmaceutical research.

**Figure 2:**
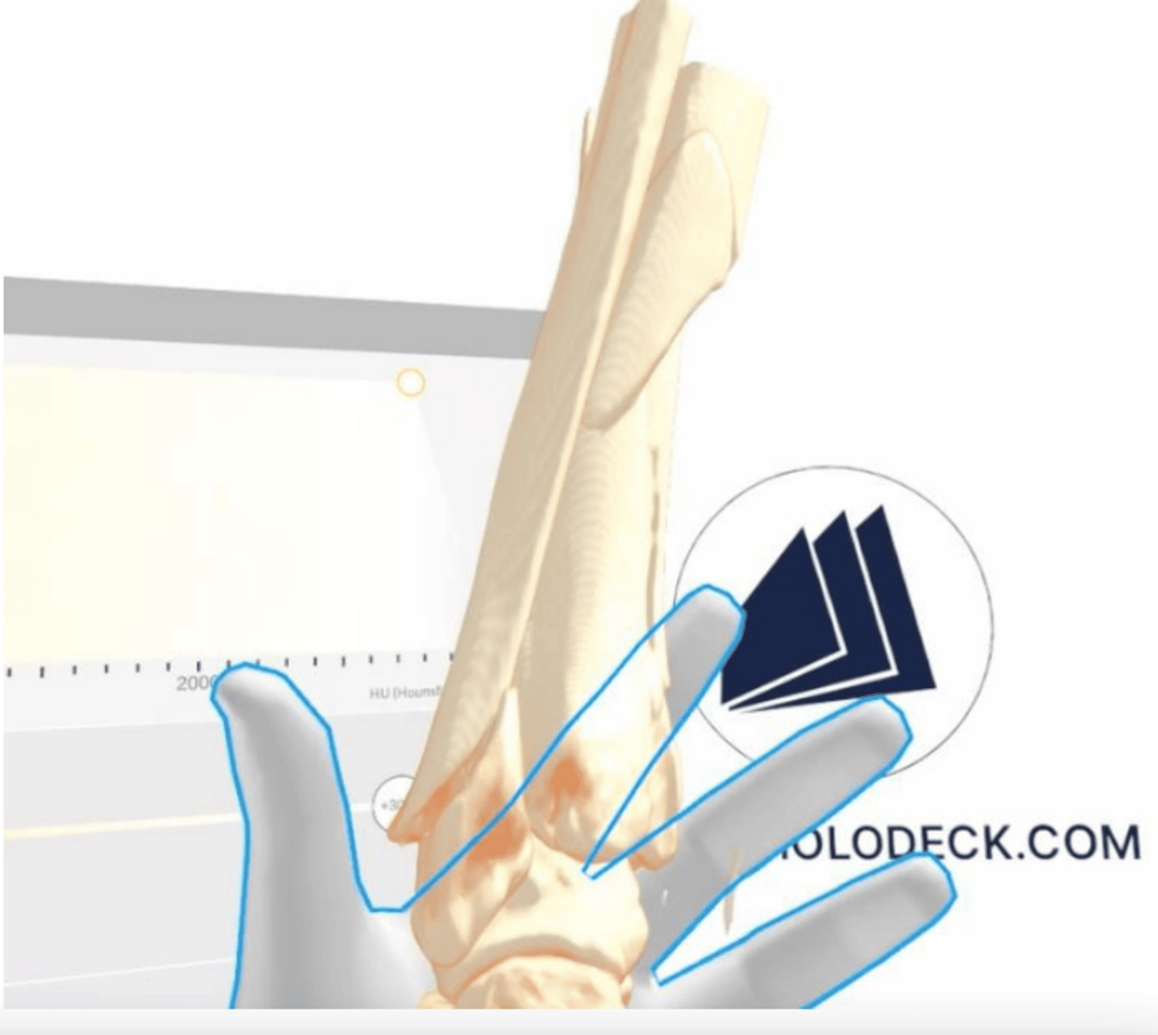
The CT image in DICOM file format from Figure 1 has been imported to MedicalHolodeck™ for visualisation and manipulation in VR DICOM: Digital Imaging and Communications in Medicine; VR: virtual reality This figure has been sourced from the "LOVE" (Lanarkshire Orthopaedic Virtual Environment) system, the in-house system at NHS Lanarkshire. Reproduced with permission from Medicalholodeck and Stryker.

An existing NHS server computer (Intel Xenon PC with free PCI expansion port, 32 GB RAM (Intel Corp., Santa Clara, CA, USA)) was upgraded with a GeForce RTX 3060 12 GB GDDR6 graphics card (NVIDIA Corporation, Santa Clara, CA, USA). This was connected to an existing monitor (BenQ 50-inch plasma screen (BenQ Corp., Taipei, Taiwan)) via a high-definition multimedia interface (HDMI) cable and to a Meta Quest 2 VR headset (Reality Labs (Meta Platforms), Menlo Park, CA, USA) via a USB-C link cable. The total cost of additional hardware was £1047.00 (£399 for the Quest 2™ Gaming Headset, £559 for the graphics card through the NHS, and £89 for the Questlink™ gaming cable). The latest VR MedicalImaging XR™ software was installed on the personal computer (PC) (through the Steam platform (Valve Corporation, Bellevue, WA, USA)), facilitating the import of patient imaging in DICOM file format to visualise images in a 3D virtual environment, which can also load .STL implant files. Various subscriptions are available to utilise Medicalholodeck®, which vary with institution, duration, and number of applications provided. For our particular institution, hardware and software costs amounted to less than £3000 for 2023-2024. Users can use controllers to manipulate images with their hands to scale to size, walk around, and examine reconstructions from multiple perspectives, as seen in Figure 2.

**Figure 3:**
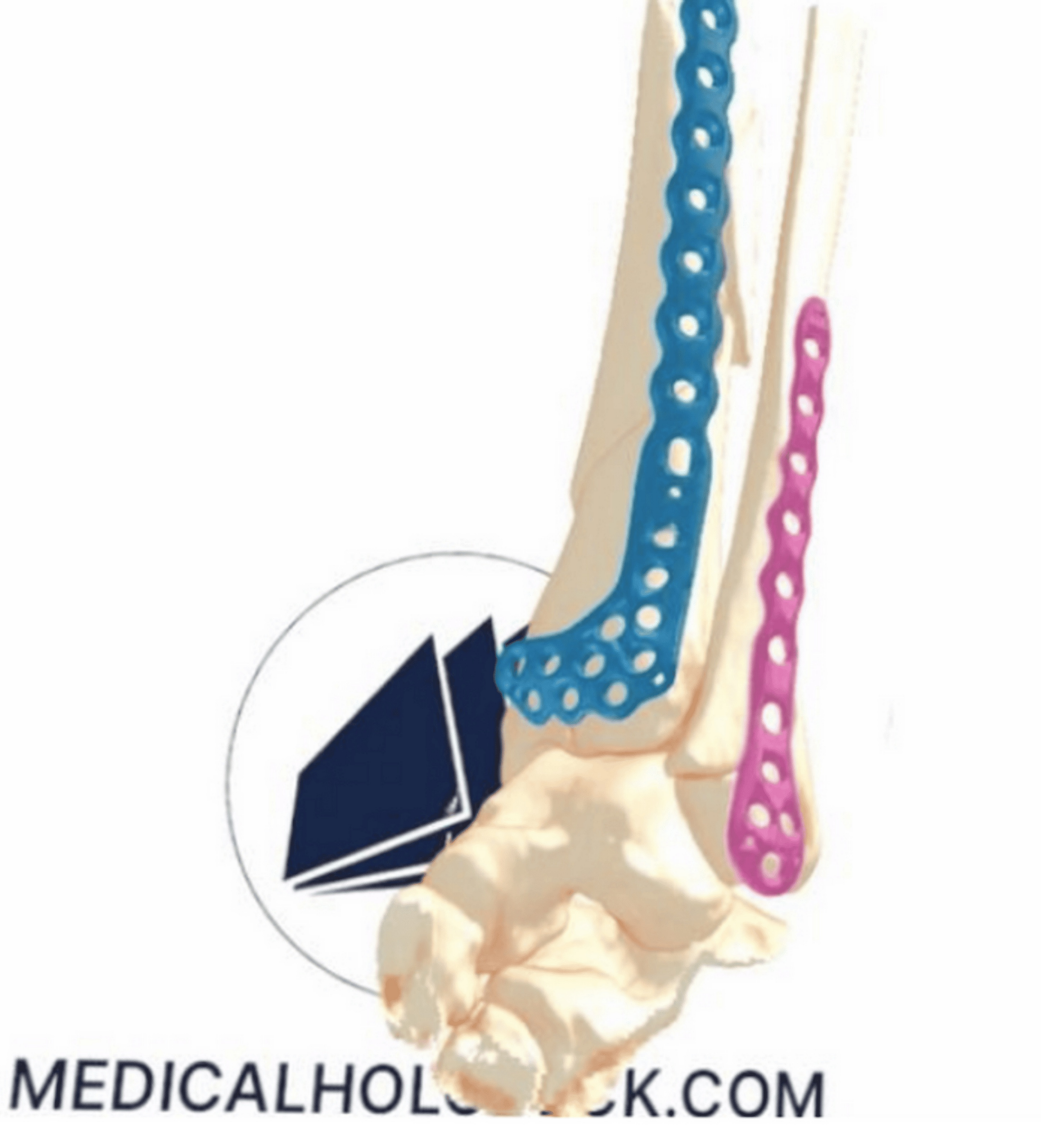
The patient-specific DICOM file from Figure 2 can be manipulated separately from .STL implant files, allowing for independent pre-operative templating and surgical planning DICOM: Digital Imaging and Communications in Medicine This figure has been sourced from the "LOVE" (Lanarkshire Orthopaedic Virtual Environment) system, the in-house system at NHS Lanarkshire. Reproduced with permission from Medicalholodeck and Stryker.

To facilitate visualisation of fractures and application of trauma plates in 3D via the VR headsets, a protocol was developed in line with local data regulations. Patient-specific images were opened with the current PACS software on a desktop PC, and imaging DICOM files were exported to be “saved” locally in a folder on the desktop PC. The VR Oculus Headset™ is plugged into the desktop PC, and "Local Questlink" was enabled, so the headset is given access to the desktop PC. VR Medicalholodeck™ software is then loaded, opening the MedicalImaging XR™ Virtual Environment. Volumetric patient-specific data were then loaded within MedicalImaging XR™, after which images could be manipulated in 3D to review fracture patterns. Implant-specific .STL files can be opened and placed within MedicalImaging XR™ to plan optimal fixation. Manipulation of both the patient 3D DICOM file and implant STL file in the same virtual space is possible with fixed scaling, as seen in Figure 3.

## Discussion

**Figure 4:**
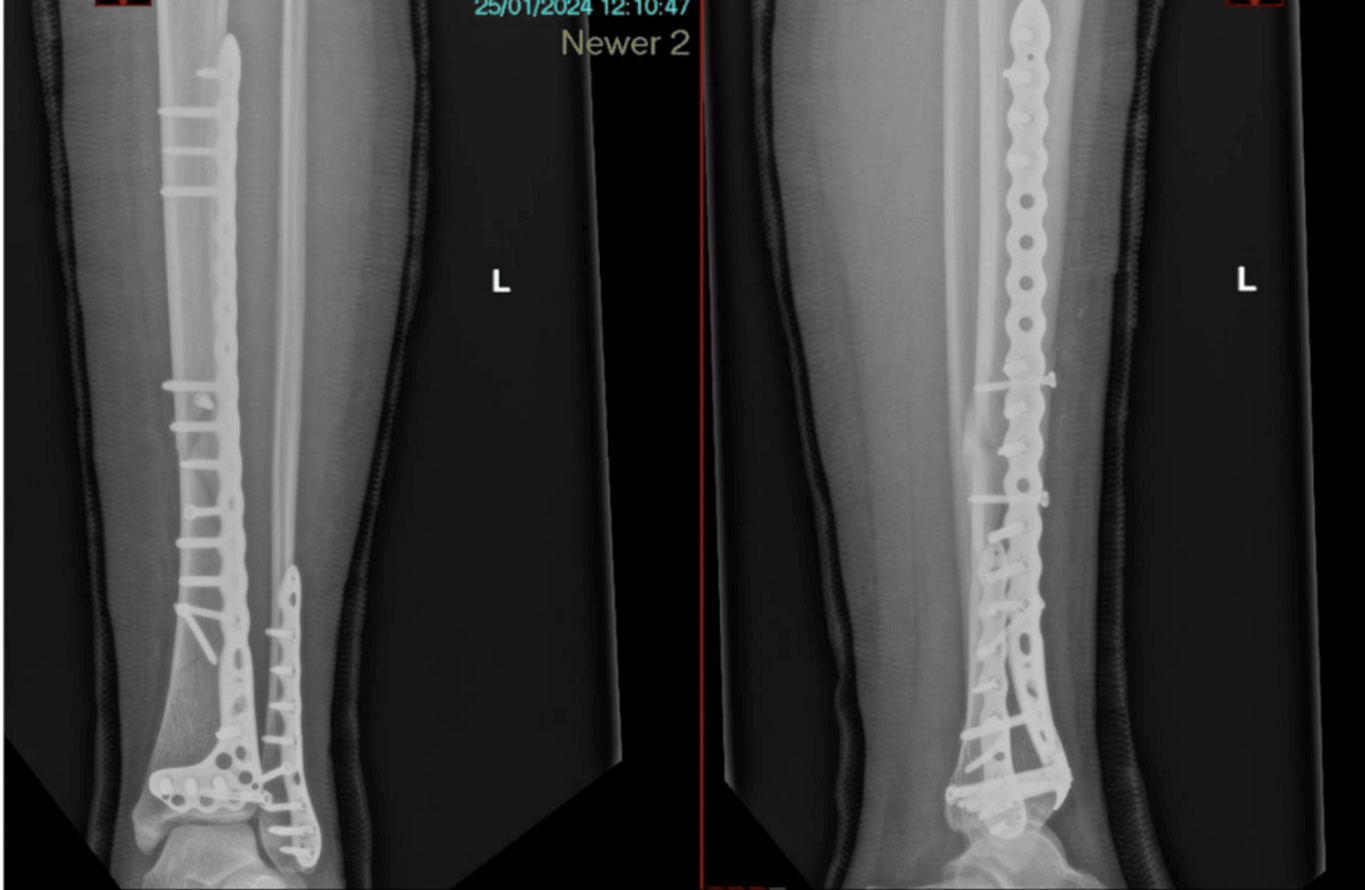
Final post-operative images following pre-operative VR templating, undertaken in Figure 3 VR: virtual reality This figure has been sourced from the "LOVE" (Lanarkshire Orthopaedic Virtual Environment) system, the in-house system at NHS Lanarkshire. Reproduced with permission from Medicalholodeck and Stryker.

To our knowledge, we are the first to describe the use of this low-cost, high-fidelity VR system using Medicalholodeck™ software, allowing visualisation of stereoscopic 3D patient images with specific orthopaedic surgical implants in the same virtual space. We have been able to demonstrate proof of concept in what we believe will be a highly useful tool to aid trauma and Orthopaedic pre-operative surgical planning and execution. This relatively low-cost system has been utilised using some additional hardware and new software, with patient-specific imaging obtained as a routine part of their clinical care .STL files of implants obtained directly from the manufacturer. We believe this has the capability to improve surgeon understanding of fracture patterns and could potentially be a useful tool to enhance patient care and surgeon performance. This system has been utilised locally to aid pre-operative planning and execution, as seen in Figure 4.

In the future, we will look to demonstrate perceived usefulness by the orthopaedic team for fracture understanding and trauma planning. It is plausible that a VR system allowing accurate templating for orthopaedic trauma surgery could be of great benefit to both surgeon and patient and may have an influence over such factors as operative time, failure of fixation, surgeon satisfaction, and patient outcomes.

## Conclusions

Orthopaedic trauma burden is increasing in volume and complexity, with error rates in surgery increasing and related patient morbidity. Incorrect use of surgical implants might be minimised through preoperative planning. Here, we present a proof-of-concept in the use of a novel VR system allowing the visualisation of patient-specific imaging with an implant .STL files, facilitating 3D trauma visualisation and planning for complex trauma.
